# Two Cases of Intestinal Polypus

**Published:** 1885-09

**Authors:** H. Boyle Runnals

**Affiliations:** Saltash, Cornwall


### TWO CASES OF INTESTINAL POLYPUS. By H. Boyle Runnals, M.R.C.S., Saltash, Cornwall.

I have published an account of these cases, and more particularly the first one, as an example of the great difficulty so often experienced in diagnosing the causes of intestinal obstruction; and it is more interesting since the thorough educating discussion upon that subject reported in the March edition of this *Journal*. It will be observed that these cases produced exactly opposite symptoms: the first one, those of intestinal stoppage; and the other, chronic diarrhœa, or rather, an inability on the part of the rectum to retain its contents.

Mrs. W., aged 45, married, and the mother of three children, came under my care twelve months ago, and was then suffering from intestinal obstruction, which I attributed, from the absence of other symptoms, to fæcal accumulation. Defecation had not taken place for six days, and as the symptoms were not of an acute nature, beyond the vomiting and a severe pain in the back, I gave her castor oil, with no effect; and three days after I was called in I administered a soapy-water enema, injecting with great force. This resulted in the discharge of a large quantity of hard fæcal matter. As the pain in the back had not ceased, and thinking it might be due to uterine displacement, I examined the vagina, and found the uterus slightly displaced downwards, which was easily remedied by a pessary, and the pain in the back diminished. I did not see her again until May 2nd of this year, when I was called in to attend her for a "facial carbuncle," which so extensively implicated the muscles of the face that she could not open her mouth, and the only means by which she could feed herself was sucking through a straw. This rendered her very weak, and the bowels again became obstructed. I was afraid to give her a purge on account of her exhausted state, and resorted again to an enema. This produced a copious evacuation of very hard offensive fæcal matter. The pain in the back was much worse now than it ever had been (I was told now that she had suffered from the pain in back for three years), but I could discover nothing to account for it. There was slight abdominal tenderness, more particularly in the left iliac region. Percussion elicited no dulness; in fact, I could not diagnose anything abnormal. These symptoms continued for a week, when one morning my attention was called to the condition of the urine. This I examined, and found it loaded with pus cells, and that it coagulated almost *en masse* from the quantity of albumen present. I thought there must be some other cause besides renal to give rise to such a state, and presuming it might be due to a vaginal discharge becoming mixed with it, I again examined the vagina, and to my surprise and horror found a gangrenous mass present at the orifice of that canal. On more careful examination, I found it was protruding from the rectum into the vagina through a large recto-vaginal fistula. I could not move it. I syringed the rectum and vagina with a lotion of carbolic acid, which produced a copious discharge of offensive pus. The syringing was repeated several times a day, and the fifth day the gangrenous mass, about the size of a swan's egg, was discharged. She now made rapid recovery, and at the present time is nearly convalescent, although an enormous recto-vaginal fistula remains for future treatment. My first thought on seeing this gangrenous mass was, that it was an "intussuscepted intestine;" but, of course, it could not be so, for she had not displayed any symptoms of it; neither, for the same reason, could it be of a cancerous nature. On examining it microscopically, I found it consisted of a fibro-mucous tissue. Now, remembering the constant pain in the back from which she suffered, and the obstinate condition of the bowels, I came to the conclusion that this mass of tissue was a "fibro-mucous" polypus, which had been attached to some part of the intestinal canal by a slender pedicle, which had ulcerated on account of the debilitated state she was in for such a long time. What else could it have been?

The other case was that of a man who was an inmate of the St. Marylebone Workhouse Infirmary at the time I was its Medical Superintendent. He told me that for four years he had suffered from diarrhœa, for which he had been unable to obtain a remedy, although he had been most assiduous in his endeavours to do so. Telling me he suffered from hæmorrhoids, I had a look at the anus externally, and found the veins much congested. Being very busy at the time, and not taking much interest in the case, I ordered him an astringent, and saw no more of him for some time, until one morning, when he again came to me, and said he was no better. *Age quod agis.* Thinking it queer that it was so obstinate to treatment, and as he told me that for the last twelve months he had a constant desire to defecate with a bearing-down pain, I attributed it to internal piles or possibly dysentery. I carefully examined the rectum, or so much of it as I could reach with my finger, and found an obstacle immediately inside the anus. It was a freely movable pedunculated mass attached to right side of rectum, about one inch from the orifice. Not having an ecraseur, I ligatured the pedicle by means of a Bellocq's epistaxis canula. I kept the patient in bed, and in four days I found the mass had separated, and with a pair of vulsellum forceps I extracted it after a little trouble. In a few days the apparent diarrhœa ceased, and I discharged him cured. It was about the size of a fowl's egg, and consisted of fibro-mucous material. Of course, what was thought to be diarrhœa was simply a loss of power upon the part of the sphincter muscles. I consider these cases particularly interesting and instructive, and they show how careful one always ought to be in making a diagnosis.

